# DNA Methylation Profiles in Whole Blood of Huntington's Disease Patients

**DOI:** 10.3389/fneur.2018.00655

**Published:** 2018-08-14

**Authors:** Maja Zadel, Aleš Maver, Anja Kovanda, Borut Peterlin

**Affiliations:** ^1^Clinical Institute of Medical Genetics, University Medical Centre Ljubljana, Ljubljana, Slovenia; ^2^Community Health Centre Ljubljana, Ljubljana, Slovenia

**Keywords:** Huntington's disease, 5mC methylation, differential DNA methylation, epigenetics, whole blood

## Abstract

Epigenetic mechanisms, especially DNA methylation, are suggested to play a role in the age-of-onset in Huntington's disease (HD) based on studies on patient brains, and cellular and animal models. Methylation is tissue-specific and it is not clear how HD specific methylation in the brain correlates with the blood compartment, which represents a much more clinically accessible sample. Therefore, we explored the presence of HD specific DNA methylation patterns in whole blood on a cohort of HDM and healthy controls from Slovenia. We compared CpG site-specific DNA methylation in whole blood of 11 symptomatic and 9 pre-symptomatic HDM (HDM), and 15 healthy controls, by using bisulfite converted DNA on the Infinium® Human Methylation27 BeadChip microarray (Illumina) covering 27,578 CpG sites and 14,495 genes. Of the examined 14,495 genes, 437 were differentially methylated (*p* < 0.01) in pre-symptomatic HDM compared to controls, with three genes (*CLDN16, DDC, NXT2*) retaining statistical significance after the correction for multiple testing (false discovery rate, FDR < 0.05). Comparisons between symptomatic HDM and controls, and the comparison of symptomatic and pre-symptomatic HDM further identified 260 and 198 differentially methylated genes (*p* < 0.01), respectively, whereas the comparison of all HDM (symptomatic and pre-symptomatic) and healthy controls identified 326 differentially methylated genes (*p* < 0.01), however, none of these changes retained significance (FDR < 0.05) after the correction for multiple testing. The results of our study suggest that methylation signatures in the blood compartment are not robust enough to prove as valuable biomarkers for predicting HD progression, but recognizable changes in methylation deserve further research.

## Introduction

Huntington's disease (HD) is a fatal autosomal dominant neurodegenerative disorder manifested by progressive impairment of motor function, cognitive decline, and various psychiatric symptoms, beginning typically after 45 years of age (1). HD is a monogenic disease and is caused by a pathological CAG triplet expansion in the *HTT* gene coding for the Huntingtin protein. Pathological expansion lengths below 40 are not always fully penetrant, and while longer lengths show some inverse correlation with the HD age-of-onset, additional environmental factors, genetics, and epigenetics likely also play a role (2–5).

Up to date, epigenetic changes in HD have been studied predominantly in the brains of patients, as well as in cell and animal models (6). In these studies, epigenetic changes have been identified to occur both in general processes such as histone acetylation and methylation (7–9), as well as in specific epigenetic changes, such as DNA methylation (10–16). Cytosine DNA methylation (5-mC) is one of the most studied specific modifications. It typically occurs at CpG sites which are enriched in regions called CpG islands (17) and is involved in gene expression silencing and regulation of splicing. Furthermore, this tissue-specific process is thought to be one of the main mechanisms regulating tissue-specific gene expression (18, 19).

The DNA methylation studies performed so far on the brains of HD patients have not been conclusive and have suggested that while 5-mC methylation in the cortex may have minimal association with HD status, its level may nevertheless be associated with the disease age-of-onset (14). This is important as intermediate-length pathological expansions are not fully penetrant in HD and the DNA methylation status may, therefore, have some predictive value in regard to the age-of-onset. Furthermore, even in the case of larger pathological expansions, which are considered to be fully penetrant, the age-of-onset and HD progression cannot be accurately predicted from the length of CAG repeat alone due to various influencing factors (3, 20, 21). Therefore, the identification of disease-modifying genetic factors for HD presents an important priority, and biomarkers from a more easily obtainable tissue than the CNS, such as whole blood, are needed for any future clinical use.

In order to identify any HD specific epigenetic changes in whole blood, we performed a whole-genome study of DNA methylation status in peripheral blood of 11 symptomatic and 9 pre-symptomatic HD mutation carriers (HDM), and 15 healthy controls.

## Materials and methods

### Cohort characteristics

Neurological status of HD patients was assessed by an experienced HD neurologist using Unified Huntington's Disease Rating Scale (UHDRS) (22) using total functional capacity score. All samples were obtained in accordance with institutional and national review boards (Republic of Slovenia National Medical Ethics Committee, Permit No. 138/03/11), and written informed consent was given by the participants. All pre-symptomatic individuals and patients were confirmed as carriers of the pathological CAG triplet expansions in the *HTT* gene. Only one HD patient received an angiotensin II receptor blocker, with the rest being drug-naïve at the time of blood withdrawal. Thirty-five samples were included in the study. Twenty mutation carriers were different in age due to pre-symptomatic (9 samples−4 males, 5 females, age 33.6 ± 7.26; UHDRS 0-3) and symptomatic (11 samples−5 males, 6 females, age 58.0 ± 15.00; UHDRS above 50) stage of the disease and 15 healthy controls (7 males, 8 females, age 38.1 ± 11.05) with no HD family history (all listed in Supplementary Table 8).

Blood samples were taken using EDTA blood collection tubes (BD Vacutainer® Blood Collection Tube) and DNA isolation from whole blood was performed using FlexiGene DNA Kit (Qiagen GmbH, Hilden, Germany), all according to the manufacturer's protocol.

The quality of DNA was analyzed using Thermo Scientific NanoDrop 2000c Spectrophotometer (Nanodrop Technologies, USA). The starting concentration of DNA for methylation analysis was 50 ng/μL. One microliters of DNA was used for the microarray analysis.

### DNA methylation profiling

Microarray methylation analysis was performed on bisulfite converted DNA using Infinium® Human Methylation27 BeadChip microarray (Illumina Inc, San Diego, California, USA), according to the manufacturer's protocol. DNA methylation datasets in matrix format were obtained by Bead Array Reader (Illumina, USA) (23).

### Bioinformatics and statistical analysis

Quality control and quantile normalization were performed using Lumi package (24). Batch effect correction was assessed by ComBat tool using non-parametric empirical Bayes statistical method (25). Statistical comparison of methylation values was performed using MethyLumi package from Bioconductor v2.8 project in R statistical environment version 2.13.1. (26). Statistical comparisons were performed between all pairs of the three groups of subjects: symptomatic HDM (S), pre-symptomatic HDM (PS), and controls (K). Additionally, methylation levels of all HDM (symptomatic and pre-symptomatic) were compared against the controls. Statistical significance cut-off value was set to *p* < 0.01 and a false discovery rate (FDR) < 0.05 to correct for multiple testing. In order to better portray actual DNA methylation differences between groups we calculated the Δ% of the average 5-mC for each position.

### Comparison of DNA methylation and transcriptomic changes in HD

In order to identify an overlap between methylation patterns and transcriptomic changes in HD in whole blood, a comparison was performed between the results of the methylation of all HDM and controls (326 genes reaching *p* < 0.01) and the 740 previously identified differentially expressed (DE) transcripts (*p* < 0.01, FDR < 0.1) in the same HD cohort (27), as well as the 15 consistent DE transcripts identified by three independent studies (27). Shared genes identified by methylation and transcriptomic comparisons were visualized using an online VENN tool (http://bioinformatics.psb.ugent.be/cgi-bin/liste/Venn/calculate_venn.htpl).

### Gene phenotype, disease, and GO annotation

Top 30 differentially methylated genes from the comparison of all HDM and controls, and 12 genes with overlapping differential DNA methylation and differential expression determined previously (27), were annotated for associated phenotypes, diseases, and GO_biological process using the online PANTHER™ tool version 12.0 (28) and the ENSEMBL Biomart tool (29).

## Results

**Figure 1:**
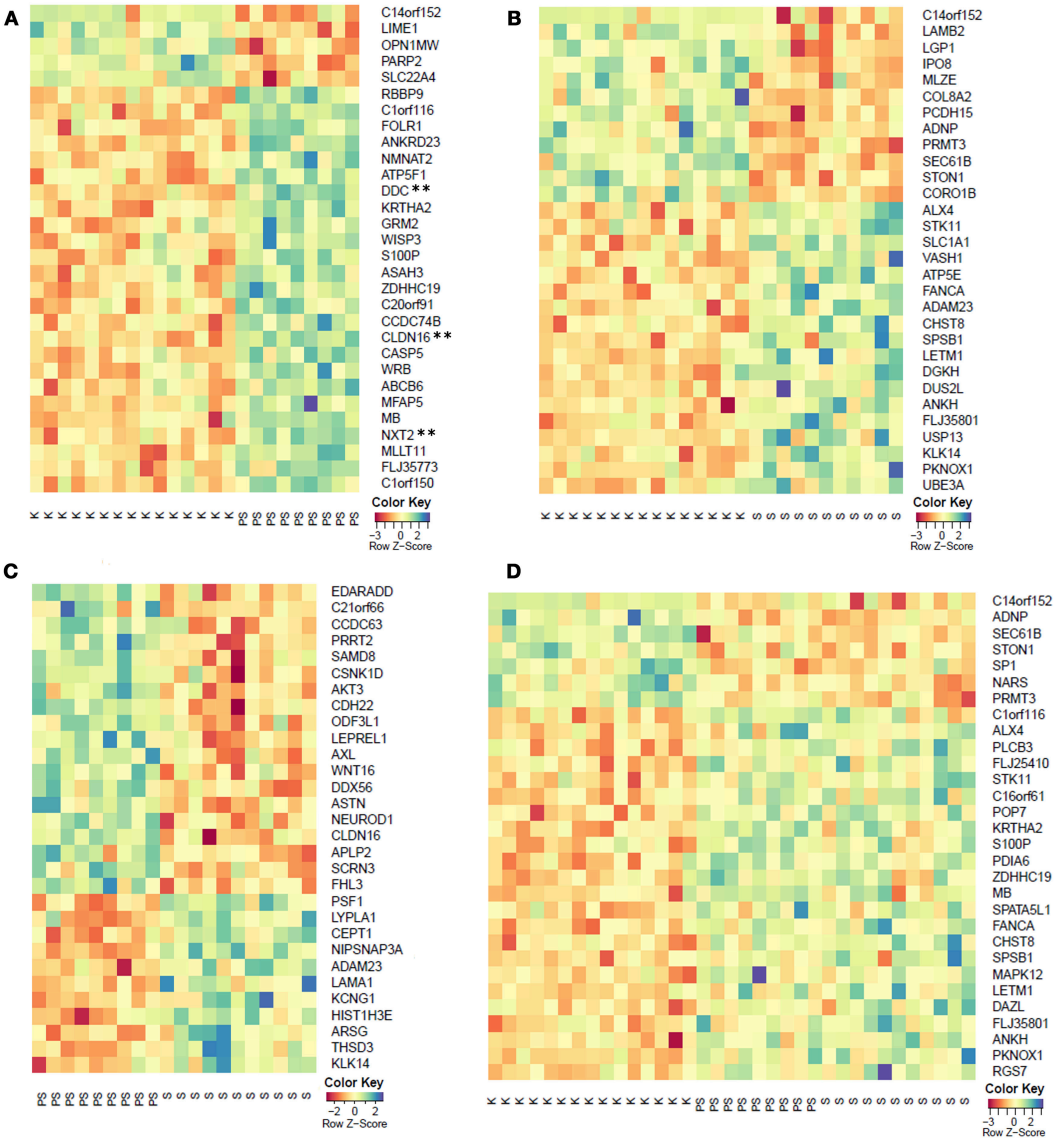
Top 30 differentially methylated genes (*p* < 0.01) from the comparison of **(A)** pre-symptomatic carriers and controls, **(B)** symptomatic HDM and controls, **(C)** pre-symptomatic and symptomatic HDM, and **(D)** pooled HDM and controls. K, controls; PS, pre-symptomatic HDM; S, symptomatic HDM. **FDR < 0.05.

Microarray methylation analysis of 27,578 CpG sites covering 14,495 genes was performed on bisulfite converted DNA from whole blood of 11 symptomatic, 9 pre-symptomatic HDM, and 15 healthy controls. Paired comparisons between pre-symptomatic HDM and controls showed 437 genes to be differentially methylated (*p* < 0.01), of which three (*CLDN16, DDC, NXT2*) retained statistical significance (FDR < 0.05) after the correction for multiple testing. Paired comparisons between symptomatic HDM and controls, and symptomatic and pre-symptomatic HDM further identified 260 and 198 differentially methylated genes (*p* < 0.01), respectively, however none retained statistical significance (FDR < 0.05). Similarly, while the comparison of all HDM (symptomatic and pre-symptomatic) and healthy controls identified 326 differentially methylated genes (*p* < 0.01), none remained significant (FDR < 0.05). The top 30 hyper- and hypo-methylated genes for each of the comparisons are shown in Figure 1 and the associated diseases/phenotypes are given in Supplementary Table 1. The complete methylation data and comparisons are provided in Supplementary Tables 3–7.

**Figure 2:**
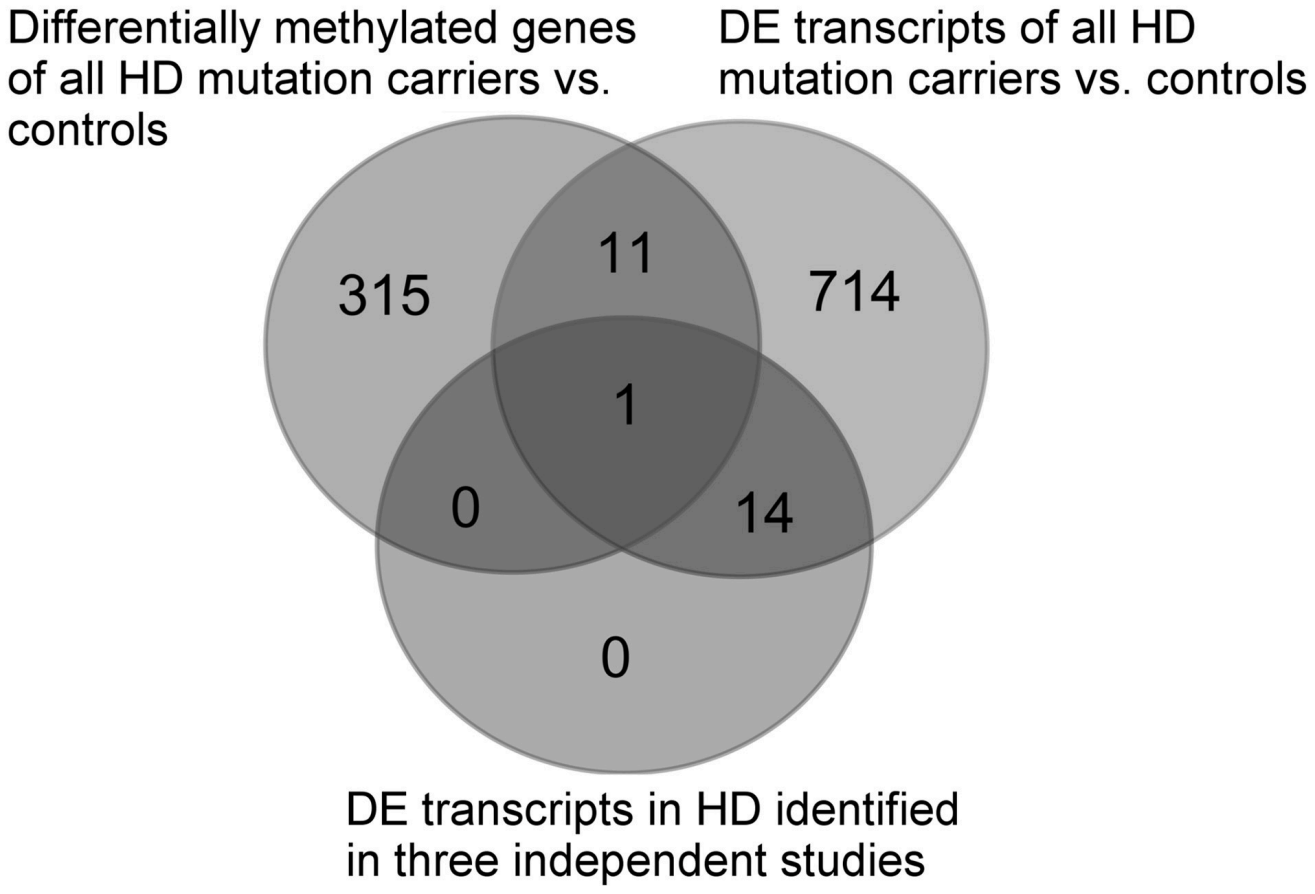
Comparison between differentially methylated genes of all HDM and controls and previously identified DE transcripts in the same cohort (27), as well as the consistently identified DE transcripts identified in whole blood by three independent HD studies (27). DE, differentially expressed.

In order to identify any global links between methylation and transcriptomic changes in whole blood in HD, we performed a comparison of the 327 differential methylated genes between all HDM and controls, and 740 DE transcripts previously identified in the same cohort (27). The comparison showed 12 genes (*FBXL5, S100P, PRDX1, COPS7B, SP1, SEC24C, PDIA6, USP5, GRAP, POP5, WRB, PCSK7*) with differential methylation and transcription in the same HD cohort (27) (Supplementary Table 2), however, none of the overlapping genes were supported by FDR in the case of differential methylation. We also compared the differentially methylated genes and DE transcripts with the 15 consistently DE transcripts identified in independent cohorts (27), and we found an overlap in a single gene, F-box/LRR-repeat protein 5 (*FBXL5)*, a protein involved in chaperonin-mediated protein folding and the innate immune system (Figure 2).

## Discussion

In our various group comparisons of CpG methylation in whole blood, we identified several hundred differentially methylated genes (*p* < 0.01), of which only three [claudin 16 (*CLDN16)*, dopa decarboxylase (*DDC)*, and nuclear transport factor 2 like export factor 2 (*NXT2*)] retained significance after the correction for multiple testing (FDR < 0.05) in the comparison between pre-symptomatic HDM and controls, but not in any of the other group comparisons. *DDC* has so far been implicated in aromatic L-amino acid decarboxylase deficiency disorder, while *CLDN16* has been implicated in hypomagnesemia, hypercalciuria, and nephrocalcinosis, whereas *NXT2* is involved in mRNA and protein transport, however, none of the three genes have been previously linked with HD. Additionally, we were unable to confirm differential methylation in whole blood, of the three genes previously observed as being differentially methylated in brain and other tissues: the adenosine A (2A) receptor gene (*ADORA2A*) (15), CCCTC-binding factor (*CTCF*) (14), or Huntingtin (*HTT)* (14, 16). This likely reflects the differences in their tissue-specific methylation and expression between blood and other tissues, as well as methodological differences in detection in the case of HTT, for which a different methylation site was examined in the original study (16).

Similarly, our comparison of the differentially methylated genes between all HDM and controls with previously analyzed DE transcripts from the same cohort (27) showed 12 overlapping genes, none of which achieved a statistically significant FDR. This may in part reflect the differences in cell populations contributing to the pool of transcripts and DNA in whole blood, however, it was an expected result given the low concordance observed between HD transcriptomic studies and the high FDR observed in the case of methylation. One of the 12 overlapping genes, F-box and leucine rich repeat protein 5 (*FBXL5*), was also detected as being DE in whole blood in HD in three independent transcriptomic studies (27). *FBXL5* is involved in iron homeostasis and protein ubiquitination, as well as the innate immune system pathway, and may therefore be involved in the systemic response to HD; however, it has not been directly linked with HD pathology. Furthermore, of the 12 overlapping genes, the four genes (*POP5, GRAP, USP5*, and *SEC24C*) showing an expected inverse correlation between methylation and RNA expression levels are involved in RNA metabolism, the innate immune response, ubiquitination, and intracellular protein transport and antigen presentation, respectively, also supporting their involvement in systemic response to HD, however, they have not been previously linked with HD and none of them achieved FDR < 0.05 in the case of differential methylation.

Although the lack of statistical significance in our study may be the result of the relatively low number of samples and the large number of simultaneously examined CpG sites (27,578), our results are consistent with recent findings showing no change in the overall or global (not site-specific) level of 5-methylcytosine in whole blood from HD patients using an ELISA test (30), as well as the recent study where the comparison between HD and control cortex samples failed to identify statistically significant differentially methylated regions (14). Nevertheless, while 5-mC methylation in the cortex was shown to have minimal association with HD status, the study found its level to be associated with age-of-onset (14).

In other neurodegenerative disorders, where epigenetic factors are also suggested to play an important role (7, 12, 30–33), there have been mixed findings in regard to global differential methylation in whole blood. In Alzheimer's disease (AD), elevated global, not site-specific, DNA methylation has been observed in mononuclear cells from whole blood (34), and correlated with the up-regulation of plasma homocysteine. Plasma homocysteine is responsible for decreased DNA methylase activity, and is an independent risk factor for AD, known to occur at disease onset (35). Similarly, global DNA methylation in blood was shown in amyotrophic lateral sclerosis (30, 36), as well as spinocerebellar ataxias 1 and 2 (30). However, in Parkinson's disease, similarly to HD, differential DNA methylation of the alfa-synuclein gene was not confirmed in blood (37), despite being shown to be differentially methylated in brain cells of patients and healthy controls (38).

Together, the recent results including our study, indicate that whole blood is unlikely to prove robust epigenetic biomarkers predictive of HD progression and age-of-onset, however, since the ELISA approach does not enable identification of specific CpG sites, while our study has, for the first time, examined the methylation of 27,578 of the estimated 28 million distinct CpG sites present in the whole human genome, it remains possible that additional differentially methylated sites may be identified through further research on larger cohorts.

## Conclusions

Our results based on the examined 27,578 CpG sites suggest that HD methylation signatures in the blood compartment are not sufficiently strong to prove as valuable biomarkers for predicting age-of-onset or HD progression, however, recognizable changes in methylation in HD deserve further research.

## Author contributions

MZ and BP conceived the study. MZ and AM collected patients/controls and performed the experiments. AM and AK analyzed data and performed the bioinformatics analyses. All authors contributed substantially to the final manuscript.

### Conflict of interest statement

The authors declare that the research was conducted in the absence of any commercial or financial relationships that could be construed as a potential conflict of interest.

## Abbreviations

5-mC: cytosine DNA methylation
AD: Alzheimer's disease
DE: differentially expressed
FDR: false discovery rate
HD: Huntington's disease
HDM: Huntington's disease mutation carriers
UHDRS: Unified Huntington's Disease Rating Scale.
